# Health in All Policies in South Australia—Did It Promote and Enact an Equity Perspective?

**DOI:** 10.3390/ijerph14111288

**Published:** 2017-10-25

**Authors:** Helen van Eyk, Elizabeth Harris, Fran Baum, Toni Delany-Crowe, Angela Lawless, Colin MacDougall

**Affiliations:** 1 Southgate Institute for Health, Society and Equity, Flinders University, GPO Box 2100, Adelaide, SA 5001, Australia; fran.baum@flinders.edu.au (F.B.); toni.delanycrowe@flinders.edu.au (T.D.-C.); colin.macdougall@flinders.edu.au (C.M.); 2 Centre for Primary Health Care and Equity, Level 3, AGSM Building, University of New South Wales, Sydney, NSW 2052, Australia; e.harris@unsw.edu.au; 3 College of Nursing and Health Sciences, Flinders University, GPO Box 2100, Adelaide, SA 5001, Australia; angela.lawless@flinders.edu.au; 4 College of Medicine and Public Health, Flinders University, GPO Box 2100, Adelaide, SA 5001, Australia

**Keywords:** Health in All Policies, equity, social determinants, healthy public policy, intersectoral action

## Abstract

Mobilising cross-sectoral action is helpful in addressing the range of social determinants that contribute to health inequities. The South Australian Health in All Policies (SA HiAP) approach was implemented from 2007 to stimulate cross-sector policy activity to address the social determinants of health to improve population wellbeing and reduce health inequities. This paper presents selected findings from a five year multi-methods research study of the SA HiAP approach and draws on data collected during interviews, observation, case studies, and document analysis. The analysis shows that SA HiAP had dual goals of facilitating joined-up government for co-benefits (process focus); and addressing social determinants of health and inequities through cross-sectoral policy activity (outcomes focus). Government agencies readily understood HiAP as providing tools for improving the process of intersectoral policy development, while the more distal outcome-focused intent of improving equity was not well understood and gained less traction. While some early rhetorical support existed for progressing an equity agenda through SA HiAP, subsequent economic pressures resulted in the government narrowing its priorities to economic goals. The paper concludes that SA HiAP’s initial intentions to address equity were only partially enacted and little was done to reduce inequities. Emerging opportunities in SA, and internationally, including the UN Sustainable Development Goals, may revive interest in addressing equity.

## 1. Introduction

Addressing the social determinants of health through intersectoral action is important in improving health equity [1] because progress depends upon health promoting policy decisions being made within sectors outside of health departments [2]. Public policies are the key levers used by governments to address fundamental social issues such as equity and inequity. Health in All Policies (HiAP) is an approach to forming intersectoral relationships and developing intersectoral policies across government to address the social determinants of health and equity [3,4,5]. Achieving equity in health is often cited as a core aim of HiAP [2,6]. 

Our understanding of the conceptual underpinning of the causes of health inequities is rapidly evolving after more than a decade of action following the Commission on the Social Determinants of Health and associated national reviews; see for example [1,7,8]. The difference between health inequality and health inequity has been an important recurring conceptual debate. Inequity means that there are systematic differences between groups that are avoidable and considered unfair or unjust, whereas inequality simply reflects a difference in outcome between groups. As such, identifying inequity involves a number of explicit value judgements [9,10,11]. 

The recent focus on the social determinants of health has led many researchers, policy makers and advocates to assume that addressing these will automatically address health inequity. However, this has not necessarily been found to be the case [7,12,13]. For example whereas the social determinants of health include living and working conditions, the determinants of health inequity include the factors that drive the distribution of living and working conditions, or the structural drivers that shape the way societies are organised, such as trade agreements and allocation of public goods [1,14]. The causes of health inequities relate to the distributive effects of the social determinants of health and the social processes that determine that distribution, such as the inequitable distribution of power and resources, poverty and discrimination [15]. Thus health policies that seek to improve health through addressing social determinants such as education, employment or access to health services, or through a focus on groups living in disadvantage, without addressing underlying causes may not improve the unequal and unfair distribution of those determinants and may perpetuate or even increase inequity [16]. 

Some have argued that addressing the social determinants of health equity requires a focus on the gradient of health inequities and “proportionate universalism”, with responses to health inequities being universal, but also incorporating proportionate, targeted actions in response to disadvantage within the population [7,17]. A gradient approach requires addressing the distribution of resources in society and also the power differentials to enable the flattening of the social gradient across society [18]. This approach is unlikely to be adopted by governments whose values would not encourage them to challenge power [19,20] and so are unwilling to take action that would provoke opposition from vested interest groups. Health is distributed predictably along the socioeconomic gradient [11,14]. The social gradient reflects data showing that health status worsens from the top to the bottom of the socioeconomic spectrum and is present in low, middle and high income countries. The social gradient in health reflects the empirical data showing that inequalities in population health status are associated with inequalities in income and social status [1,14,21]. There is an incremental change across a population where a little more wealth gives a little more health, resulting in a gradient between the most and least disadvantaged. An increasing body of research suggests that the steepness of income inequality gradients affects overall population health and wellbeing [22,23]. As a result, inequities affect all members of society (by, for example reducing social cohesion), and addressing the social gradient and reducing health inequities benefits the whole population [18,24].

Significant health inequalities persist in Australia despite it having one of the highest life expectancies in the world. The Australian Institute of Health and Welfare [25] found that life expectancy for males and females born in 2014 was 80.3 years and 84.4 years, respectively. However the social gradient in Australia means that those living in richer areas live longer, with a difference between the highest and lowest socioeconomic areas being about three years. There is also a social gradient in Australia between cities and regional and remote areas. While 29% of Australians live in regional and remote areas, deaths in these areas accounted for almost 38% of premature deaths in 2011–2013. Most glaringly, while health outcomes for Australian Indigenous people have improved in recent years in a number of key areas, there continues to be a 10-year gap in life expectancy between Indigenous and other Australians and they continue to experience greater health disadvantage [25]. Table 1 shows the inequality rate ratio for Australia and South Australia, and the widening gap in inequalities between South Australia and Australia over this time.

Australia is a federated nation. State governments in Australia are limited in the extent to which they can address inequity. A redistributive taxation system and universal health care system (Medicare in Australia) are national responsibilities of the federal government. However there remains potential for state governments to do more to address inequity within the scope of their responsibilities.

### The South Australian Approach to Health in All Policies

Various models of Health in All Policies (HiAP) have been implemented in many countries and regions; see for example [27,28,29]. In South Australia (SA), HiAP has been implemented since 2007 by the SA Health Department and the central agency of the SA State Government, the Department of the Premier and Cabinet. HiAP was introduced to SA as a key recommendation in the final report of Adelaide Thinker in Residence, Professor Ilona Kickbusch [30], with the explicit intention of addressing the social determinants of health and health equity through intersectoral action across government agencies. It sought to do this using the authorising structure of the State Government’s *South Australia’s Strategic Plan* (SASP) as the key vehicle through which intersectoral action would be used to address the social determinants of health [3,4]. 

The South Australian HiAP approach involves working with other sectors on cross sector policies to improve population health, wellbeing and equity while also addressing the other sector’s core business (with the intention to produce co-benefits) [31]. However stakeholders and policy makers from government sectors may have different perceptions of what health inequities are, why (and if) they are important, their causes, how they should be addressed and whether, politically, it is important to address them [18]. The different ways that health equity is understood and problematised mean that equity is often a contested or misinterpreted concept within the context of intersectoral activities [32].

Using the South Australian experience of HiAP as a case study, we employ institutional theory to consider the role that institutions, ideas and actors have played in determining the extent to which health equity has been able to be addressed as part of HiAP activities. Institutional theory was selected because it provides us with a lens of analysis through which to consider the underlying factors explaining the fate of equity, and to pay close attention to the role of policy actors and the ideas relating to equity. This paper builds on the existing literature on HiAP by considering the extent to which the articulated aim of reducing health inequities through healthy public policy has been promoted and enacted through a HiAP approach. Using institutional theory, we consider the reasons for and implications of a drift in the focus of HiAP work away from equity as a goal of HiAP, to HiAP becoming predominantly viewed as a process to facilitate joined-up policy across the SA Government. 

While there is a substantial literature on HiAP’s approach and aims internationally (see for example [27,33,34]), its effectiveness in reducing health inequities has not been addressed. This paper contributes to understanding the problems and possibilities for policy makers using a HiAP approach to address the social determinants of health equity. 

## 2. Materials and Methods

The data that inform this paper were collected over a period of five years (2012–2016), as part of a mixed methods retrospective and prospective evaluation designed to address the broader question “Does a Health in All Policies approach improve health, wellbeing and equity?” This paper draws on data from the research, including an analysis of documents that provided the underpinning authorisation and priorities for SA HiAP, a series of detailed research case studies of HiAP Health Lens Analysis projects and subsequent adaptations of the HiAP model, 144 interviews and two workshops, and a log of observations monitoring and documenting the changing political and economic contexts in which HiAP operated. 

### 2.1. Analysis of Key Documents

Five key public documents were identified as articulating the priority foci for HiAP in SA. Identification occurred during discussions at research team meetings and with policy actors who are also investigators on the project, as well as through analysis of the interview and workshop data. 

In 2007, prior to the implementation of HiAP in SA, ten principles were agreed upon to underpin the South Australian approach, and were documented as *Health in All Policies: the 10 Principles* [35]. Four other key priority setting documents reflected the SA Government’s shifting strategic agenda over the time of the research project and determined to a large extent the focus of HiAP activity, including:

- *South Australia’s Strategic Plan* (2007 version) [36]
- *South Australia’s Strategic Plan* (2011 version) [37]
- *Seven Strategic Priorities* (2011) [38]
- *Ten Economic Priorities* (2014) [39].

Analysis of these key documents considered the relative focus on equity (as described earlier and defined by Whitehead [9]), and on intersectoral collaboration in the documents’ aims and targets, and how this influenced the SA HiAP approach. In considering the extent of the documents’ focus on equity, the analysis also examined the way equity was defined: in relation to addressing the needs of vulnerable groups; access to services; and levelling up the social gradient [15]. Document analysis was supported and accompanied by interviews with key actors about the changing political context in which HiAP was operating in South Australia, and their experiences and assessments of the HiAP initiatives. 

### 2.2. Case Study Analysis

In this paper we also draw on five detailed case studies of Health Lens Analysis projects undertaken as part of the research on the effectiveness and implementation of SA HiAP. Health Lens Analysis provided the methodology for the SA HiAP approach and was a means for HiAP to assess the contribution of different sectors to the social determinants of health, and to secure other sectors’ engagement with them in healthy public policy [40]. In conjunction with analysis of the interviews with participants involved in these Health Lens Analysis projects, we analysed publicly available documentation, including project proposals, final reports and material outputs and resources produced during the projects. 

Additionally, we analysed subsequent stages of HiAP that followed its initial phase of Health Lens Analysis projects. These subsequent stages included the systematization of HiAP under the new *South Australian Public Health Act 2011*, as well as two cross-sector organisational change projects, called 90-Day Projects, led by the Health Department, with key responsibility held by HiAP staff and the participation of a number of other government agencies. The project aims, final reports and outputs from these two projects were analysed to determine the extent of their focus on equity. 

The case study method contributed in-depth, practical and detailed investigation of the application of the HiAP approach and its likely equity outcomes [41].

### 2.3. Semi-Structured Interviews and Program Logic Workshops

One hundred and forty four (144) face-to-face or telephone interviews were undertaken with policy actors from the Health Department (*N* = 53) and partner agencies (*N* = 51), local government actors (*N* = 31), political actors (*N* = 4) and academic researchers (*N* = 5), who had some role in the SA HiAP initiative. The respondents were asked about the changing context in which the HiAP approach was being implemented, and the experiences and perspectives of participants in relation to a selection of case studies of HiAP Health Lens Analysis projects and subsequent government initiatives that reflected the evolution of the SA approach to HiAP. 113 of these interviews focused on respondents’ understandings of population health equity and how it related to the work of their agencies, the SA Government’s policy agenda and HiAP; the changing context in which HiAP was operating and how this affected its focus on equity; and a series of five case studies of HiAP Health Lens Analysis projects and their focus on equity. The other 31 interviews related to a specific case study of local government actors’ experience of their new legislated responsibility to undertake regional public health planning, reflecting the systematisation of HiAP beyond a Health Lens Analysis project-based approach. 

The interviews were undertaken by six researchers experienced in qualitative interviewing and guided by interview schedules. The researchers who undertook the interviews were Chief Investigators and the Project Manager for the study, reflecting the fact that many of the interviews were undertaken with senior public sector executives. Interviews were recorded and transcribed verbatim, and averaged 38 min in length, ranging from 10 min to 1 h 35 min.

In mid-2013 two workshops were held to develop a Program Logic Model to demonstrate the expected causal pathways showing how and why the HiAP program was predicted to achieve anticipated changes in health, wellbeing and equity [4]. Workshop participants were asked about the factors that led to HiAP being introduced in South Australia, and the assumptions, activities and processes that underpinned HiAP implementation, as well as the short and longer term goals of HiAP. Both workshops lasted 4.25 h. The first workshop involved nine policy actors from non-health sectors and the second involved 16 policy actors from within the Health Department. Both workshops were recorded and transcribed verbatim for collaborative analysis by the research team. These workshops provided rich sources of data on key aspects of the SA HiAP approach, including about equity and the changing South Australian context in which HiAP operated [42].

Thematic analysis of the interview and workshop transcripts was conducted using the qualitative analysis software NVivo 11. Following the initial round of open coding, five research team members (four of whom had also conducted interviews for the study) undertook collaborative, selective coding [43], and themes from the data analysis were developed and debated during regular research team meetings. In relation to this paper these meetings have built understanding of the way that equity has been conceptualized and problematised in HiAP activities, and of the implications for HiAP’s capacity to influence equity in South Australia. Team meetings were held weekly during the data collection phase of the research (from 2012 to early 2016) and subsequently changed to fortnightly meetings at the conclusion of data collection in 2016, and to 3 weekly meetings in 2017.

### 2.4. Ethics Approval

All data collection activities received prior approval from the Flinders University Social and Behavioural Research Ethics Committee (project no. 5518, approved 29 February 2012); and the SA Health Human Research Ethics Committee (project no. HREC/12/SAH/74, approved 7 November 2012). Informed consent was provided by all participants in the research prior to their interview or participation in a workshop. 

### 2.5. Theoretical Approach

In this paper we use institutional theory to build understanding of the extent to which health equity as a key policy idea influenced policy through the SA HiAP approach. Cairney explains that institutional theory uses the framework of institutions (including structures, standard operating procedures, norms, rules and mandates), ideas (ranging from world views, ideology and principled beliefs, to policy content) and actors (individuals, organisations and networks) to understand what leads to or constrains policy change [44]. Similarly, Howlett, Ramesh and Perl note that institutions are multifaceted and function to provide stability, being relatively resistant to change [45]. Scott explains that institutions comprise regulative, normative and cultural-cognitive pillars that combine with associated activities and resources to bring stability and meaning to social life [46]. Institutions shape or constrain political behaviour and influence action through shaping the interpretation of problems and constraining the choice of solutions that are possible for policy actors. In their discussions of institutional theory, both Koelble [47] and Cairney [44] note that individual and organisational actors pursue their interests in the context of institutions, which include the underlying rules and norms which shape actors’ preferences, goals and decision-making processes. In seeking to explain institutional change, Marsh notes the dialectical and interactive relationship between institutions and ideas, where institutions can constrain and shape actors’ ideas and choices, and new ideas that may have been transferred from other settings can also affect institutions by transforming agents’ perceptions of their interests and thus creating possibilities for new ways of thinking and acting [48]. Deinstitutionalisation arising from, for example, changing values and norms, can be a result of a number of destabilising factors, including new ideas (such as in relation to gender roles, race discrimination or organisational structures) which can disrupt the existence of institutions, resulting in institutional change so that previous norms and attitudes lose traction. Ideas, when embedded, and constructed for example as new rules or norms, can also form new institutions [46,49]. While equity has been refined and developed as an idea and achieved greater profile through the WHO Commission on the Social Determinants of Health [1] and subsequent reviews and debate, it has been challenging for the ideas of equity and reducing inequalities to become embedded as a new institution because of the institutional dominance of the entrenched neo-liberal ideas of the free market, privatization of public goods, individuals as consumers rather than citizens, and reduced state intervention, amongst others [50,51].

## 3. Results

First, we present a document analysis to show how HiAP’s focus on equity was diminished in South Australia as a result of shifting government policy priorities. We then present a series of research case studies of HiAP activity, considering the extent to which each of these has focused on equity. Finally, we present interview data to show how equity has been understood and problematised by policy actors involved with HiAP, and how they understand the positioning of equity in regard to the business of government agencies, including within the context of competing political agendas, and we consider the implications of a co-benefits approach for an explicit focus on equity. Findings from our documented observations of the changing political and economic context in which HiAP operated are distributed throughout and inform the presentation of our results.

### 3.1. How Equity Diminished on the Government Policy Agenda—Evidence from Document Analysis

Key strategic policy documents provided the direction for whole of SA Government priorities and action during the period of this study and thus are central institutional components that shaped decisions about the policy issues to be pursued by HiAP. The five key strategic documents that provided the basis for the operation of HiAP and identified the key priorities that were driving government activity during the term of the research highlight this shift. Table 2 summarises analysis of these documents according to the presence of equity considerations, and the documents’ problematisation of equity, as well as their focus on intersectoral collaboration. In Table 2 green shading indicates clear evidence of an equity focus, orange indicates limited evidence, and red indicates no evidence of an equity focus.

#### 3.1.1. Health in All Policies—The 10 Principles 

The HiAP initiative was implemented in South Australia in 2007 following the Residency of Professor Ilona Kickbusch as Adelaide Thinker in Residence, whose report to the SA Government [30] recommended the establishment of a HiAP approach in South Australia and linked it to *South Australia’s Strategic Plan* (SASP); for more details see [3]. The 10 Principles for HiAP [35] were developed by Kickbusch and public servants at the conclusion of her Residency. 

The focus of the ten principles is predominantly on intersectoral collaboration. Five of the ten principles concern working across government/sectors to improve health, for example:

*Principle 2.*  Recognises that health is an outcome of a wide range of factors—such as changes to the natural and built environments and to social and work environments—many of which lie outside the activities of the health sector and require a shared responsibility and an integrated and sustained policy response across government.

*Principle 6.*  Acknowledges that efforts to improve the health of all South Australians will require sustainable mechanisms that support government agencies to work collaboratively to develop integrated solutions to both current and future policy challenges.

Principle 4 explicitly identifies the need for HiAP to focus on equity:

*Principle 4.*  Recognises that the impacts of health determinants are not equally distributed among population groups in South Australia and aims at closing the health gap, in particular for the Aboriginal peoples.

A focus on equity was therefore evident as an underpinning principle of HiAP at its establishment, even though it was not the dominant focus. The dual aims of HiAP are evident in these ten principles, which focus on improving health, wellbeing and equity on the one hand (an outcome-focused goal), and working collaboratively across government to progress integrated solutions to complex policy problems (a process-focused goal) on the other.

#### 3.1.2. South Australia’s Strategic Plan (2007 and 2011 Versions)

Kickbusch’s Adelaide Thinker in Residence final report to the SA Government identified the HiAP approach as a strategic intersectoral mechanism that would support the targets of *South Australia’s Strategic Plan* (SASP) being met [30]. SASP was an evolving document with regular built-in consultative reviews that described the government’s values, priorities and targets for South Australia. An equity focus is evident in the SASP targets and can be seen to have been developed and refined between the 2007 and 2011 versions of SASP [36,37]. Examples of SASP equity-focused targets are included in Table 3.

Thus there is evidence of a shift to a stronger focus on equity between the 2007 and 2011 versions of the SASP. While including a focus on equity within the SASP targets, the process for addressing these targets and the accountability for them was strongly oriented towards breaking down silos between SA Government departments and working across sectors to address the priority targets in the Plan. Individual agency chief executives were allocated responsibility for reporting on progress on specific targets (and this responsibility formed part of their performance agreements) but there was an expectation that agencies would collaborate in working to achieve them. SASP reflected a strong government agenda of working across sectors and silos to address the most complex and challenging ‘wicked’ policy issues [52]. SASP provided the authorising environment for HiAP action across government on both equity and intersectoral collaboration, giving HiAP the mandate and authority to approach and work with other sectors on priorities that were related to the SASP targets [4]. Despite this, in practice the links to the SASP targets could appear quite tenuous in the resulting HiAP Health Lens Analysis projects.

#### 3.1.3. The 7 Strategic Priorities

Later in 2011, following a change in State Premier, the 7 Strategic Priorities [38] were introduced and overlaid, but did not replace, the SASP. At the time of our research, SASP still had currency, despite some confusion evident among our interview respondents about the status of SASP following the introduction of the 7 Strategic Priorities. The explanation given by the SA Government for the introduction of the 7 Strategic Priorities was that they provided a “sharper short term focus” that supported and complemented the achievement of the longer term SASP priorities [53]. However, they lacked the detailed targets specified in SASP. 

In the 7 Strategic Priorities, the strategic priority “Every chance for every child” includes the following statements in relation to universal access to services for children and families:

All children can access high quality, affordable child care and preschool offered by trained staff using a rigorous curriculum.

Schools are community hubs for services aimed at supporting families and children from the time they are born. All families have access to a Children’s Centre in their local area [38].

This priority provides evidence of an equity intent in relation to children, in particular in their early years. Other strategic priorities identified in the 7 Strategic Priorities document lacked a focus on equity.

Despite not being explicitly reflected in the 7 Strategic Priorities, the government continued to maintain a focus on intersectoral collaboration to address complex policy problems at this time. This was evident in a number of new initiatives that overlapped with the intersectoral goals of HiAP, including the establishment of Change@South Australia, discussed below. The continuation of a focus on intersectoral collaboration allowed HiAP to continue to justify its relevance and value to achieving the government’s priorities despite signs of a drift in the government’s focus away from equity as an underlying principle for policy action.

#### 3.1.4. The 10 Economic Priorities

The drift away from a policy focus on equity became more pronounced with the State’s economic downturn. In 2013 South Australia experienced severe economic difficulties following the failure of an anticipated mining boom to eventuate and the decline of manufacturing in the state. In response to the worsening economic climate, the government introduced its 10 Economic Priorities [39] in 2014 with a strong focus on job creation and economic development. These economic priorities did not mention equity. 

Parallel to the 10 Economic Priorities, the government continued to focus public sector activity on intersectoral collaboration, or joined-up policy to address complex issues. This was evident in the continued support for the Change@South Australia unit, established in 2012 within the SA Government Office of Public Sector Reform. The Change@SA unit sought to drive public sector culture change to increase innovation and intersectoral collaboration to solve policy problems requiring joined-up responses. 90-Day Projects (discussed further later) were one mechanism the unit used to achieve these goals.

As explained earlier, equity was evident (although not dominant) in the language of the 10 Principles for HiAP, however it progressively slipped from SA Government documents that identified and articulated the government agenda and priorities. With this shift away from a focus on equity in government priorities, HiAP became increasingly seen by policy actors in South Australia as a catalyst for collaboration and a useful process for achieving joined-up policy to address complex issues (which could still include population health and wellbeing), rather than an initiative to improve equity through intersectoral policy development. 

### 3.2. Case Studies

Table 4 provides an overview of the research case studies, including the Health Lens Analysis projects, the two 90-Day Projects, and HiAP’s role following the commencement of South Australia’s new public health legislation. It is evident from the overview provided in Table 4 that, while each of the case studies analysed for this research varied in the extent to which it focused on equity, there was an overall trend away from an equity focus and towards a predominant focus on intersectoral collaboration. In Table 4 “√” indicates evidence of health equity; “X” indicates no evidence of health equity; and ‘?’ indicates insufficient public information available to make a judgement.

The *South Australian Public Health Act 2011* commenced in 2013, and was followed by the production of the first State Public Health Plan, and the commencement of local government regional public health planning, along with the appointment of a number of Public Health Partner Authorities.

The Health Lens Analysis projects varied in the extent to which they incorporated equity. Similarly, while the new public health legislation itself included equity as a core principle, the focus of its implementation has been, in the case of regional public health planning, more generally on the local social determinants of health rather than equity, and in the case of establishing Public Health Partner Authorities, has been on building collaborative relationships with other agencies. The intent of these relationships is to contribute to key priority areas within the State Public Health Plan. For example, to support implementation of a component of the State Public Health Plan on stronger and healthier communities and neighbourhoods and in line with its own planning priorities, the Planning Department has signed a Public Health Partner Authority agreement with the Health Department with the aim to work with Health and local government to provide quality green public open spaces to support people being active, strengthen their contact with nature, and provide places for them to relax and interact. The population health and equity intentions and outcomes of these collaborative relationships are not yet known. 

90-Day Projects were an initiative of Change@SA that commenced in 2012 and have been described as a “key method of engagement” with a focus on cross agency collaboration to address complex problems that multiple agencies have an interest in resolving. This approach brings together agencies to work intensively and collaboratively to find innovative solutions over a time-limited period of 90 days. The focus of these projects varied widely (for example: innovations in social housing, cultural safety in the workplace, and greener materials in road construction). The two 90-Day Projects led by HiAP focused on building public sector capacity to undertake joined-up policy delivery (collaboration); and developing a whole of government statement positioning South Australia as a State of Wellbeing. This statement included equity as part of the definition of wellbeing but equity was not reflected further in the final document. The two 90-Day Projects were not specifically focused on health equity, but rather on breaking down silos and working collaboratively across government and with other agencies. 

### 3.3. The Role of Equity in SA HiAP—Respondents’ Assessments

Following discussion of the policy environment and analysis of specific aspects of HiAP’s work we now turn to consider the respondents’ overall assessment of the fate of equity on the HiAP agenda. In this section we consider how South Australian public sector interview respondents perceived equity and its role in the business of government agencies, how HiAP’s focus on equity was undermined by competing political agendas, and the implications of privileging relationship maintenance and co-benefits in order to facilitate partner agencies’ engagement and collaboration with HiAP. 

#### 3.3.1. The Problematisation of Equity—How Is Equity Perceived across the Public Sector?

Only a few interview respondents readily understood health equity terminology, and many sought clarification from the interviewers before answering related questions. This is likely to be a result of the various professional and disciplinary terminologies and conceptual frameworks used between sectors, which mean that multiple meanings may be attached to the concept of equity. However, following the provision of explanations about the meaning of *health equity*, our data show that health equity was most commonly understood among both respondents from SA Health and those from other participating agencies as being about a focus on vulnerable groups, and often also about improving access to services for these groups:

…you always have to have a bit of a safety net for people who are unable to make those decisions for themselves or there are circumstances which limit their ability to participate…

(Interview 11, political actor, 2013)

…like they (Planning Department) talk about it (equity) for things like accessibility, you know, like we’re about and if you look across (the Planning Department) it will all be about having the streets and footpaths that are, and buildings that are accessible for all…

(Non-health sector Program Logic Model Workshop, 2013)

The understanding of equity as a gradient, relating to “levelling up”, was much less commonly discussed by respondents, and where mentioned, was most often the interpretation applied by respondents from the public health section of the Health Department.

…the importance of fairly distributing and sharing health resources across populations…

(Health sector Program Logic Model Workshop, 2013)

…broader economic development and raising incomes could be seen as something that tries to lift the wellbeing or equity of the community more broadly, but that’s probably not an immediate and certainly an indirect process.

(Interview 14, governance sector, 2013)

The consistent exception to this limited reference to equity was in relation to “closing the gap” for Aboriginal people, where there seemed to be a much greater recognition of addressing the inequities between Aboriginal and other Australians. However this still fails to address the role of socioeconomic position and its impact on health across the population [15]:

There’s not a strategic focus within SA Health on the need to address health equity, with the exception of Aboriginal health and wellbeing, but not more broadly in terms of other population groups…

(Interview 07, health sector, 2013)

Closing the health and life expectancy gap to reduce disadvantage for Aboriginal people is a policy issue of long-standing concern for public sectors across Australia, and so it is not surprising that understanding of equity as closing the gap for Aboriginal people was more familiar and generally recognised by policy actors.

#### 3.3.2. Respondents’ Perceptions of the Role of Equity in the Business of Government Agencies

Individuals from agencies participating in HiAP activities expressed mixed views about the role of equity in the work of their agencies. Some said that equity was in the foreground of their agency’s focus, but others did not feel this was the case:

…I don’t think that that idea of health equity would be a concept that was familiar in an organisation like ours.

(Non-health sector Program Logic Model Workshop, 2013)

Many respondents said that equity, social justice and fairness are values that underpin the work of the HiAP team. This value base was also recognised by HiAP team members:

…there needs to be an underlying value about fairness and equity and wanting to ensure that people’s lives are better, so not accepting the status quo and whilst recognising the political landscape, you know, there’s a vision and an ambition to make things better.

(Interview 07, health sector, 2013)

…all of the team have that really strong focus on social justice and inequity and a desire to close or address some of those issues, close the gap, address the issues.

(Interview 35, health sector, 2013)

However, despite their articulated commitment to an underpinning value of equity, the HiAP team found that the term equity “did not resonate” with other agencies and so a conscious decision was made not to use equity language in their work with partner agencies. HiAP team members said that despite this they still sought opportunities to progress an equity agenda without this being explicitly articulated:

I don’t think it’s explicit in the work that we do, even in Health in All Policies. The term equity can be a bit challenging for other government agencies (…) Look, I think when we talk about doing projects we don’t talk about health equity or equity more generally. I think it’s just sort of implicit in what we do.

(Interview 30, health sector, 2013)

Part of the reluctance of the HiAP team to talk about health equity to partner agencies was because they did not find that the term had meaning for other agencies. Members of the HiAP team said that the term was perceived as “health jargon” or “health language”:

…a commitment to health equity is part and parcel of what we do but we don’t put it at the forefront because it has—I don’t think it has utility.

(Interview 31, health sector 2013)

…it wasn’t a “oh you can’t talk about that”, it was probably more of a “it’ll make more sense to people if we talk about it this way” or “if we come in talking about health equity, what’s the agriculture department going to think? That’s not kind of everyday language. But if we talk about the populations that they serve and their client base or their customers, that kind of makes more sense”. I think maybe some of it was about the “what’s going to make sense?” because we kept thinking what’s going to make sense when we talk to them?

(Interview 83, academic, 2014)

There was a clear acknowledgement by policy actors from both the Health Department and partner agencies that promoting equity is the proper role of government. Some respondents explained that equity was articulated rhetorically in government policy, but that this was not then evident in the implementation of government strategies:

…this and just about every other government would certainly claim to be committed to equity, it’s a question of what that actually means in the light of the cut and thrust of the politics of the day and the financial constraints that are upon them.

(Interview 73, health sector, 2014)

…there was a rhetorical commitment versus functional commitment and, you know, the clear thing is you could find any number of policy documents, whether it’s produced by us (the Health Department) or by another state government health centre service... but when you dig into it, it either quickly cascades into care or functionally it has minimal resources attached to it, so again I challenge that there is indeed a linkage. What there is, is a small space. Because that rhetoric is there, that means there has to be at least something that they can point to.

(Interview 31, health sector, 2013)

We found some discomfort, in particular among health respondents, about the lack of emphasis on equity. Respondents spoke often about why they could not progress an equity agenda, but there was less discussion about what they could do to address equity.

Equity’s never been a funding indicator, it was always a very, very long term outcome of which systems need to be built and put into place to achieve, of which does it fit into a political cycle and environment.

(Interview 74, health sector, 2014)

I guess the difficulty for this agency is that demands on us in terms of the way that economic development’s played out, equity is probably not a key driver. I mean there’s a lot of rhetoric about trying to create a level playing field, so focus just on the fundamentals of the economy (…) I mean I think that’s understood by the agency but to the extent to which it is actually undertaken by the agency is another matter and we certainly don’t control the levers for that.

(Interview 16, primary industries sector, 2013)

This latter quote highlights how the equity agenda is subordinate to the dominant economic development political and policy agenda. 

#### 3.3.3. Focus on Equity Was Undermined by Competing Political Agendas

Early discussions and planning for HiAP included a focus on addressing health equity, at least at a rhetorical level. However, this focus became less evident over time, as the focus of HiAP’s activity moved from planning to implementation, and with a decline in the state economic context and consequently a shift in government policy priorities. 

Equity is generally not seen as core business in SA Government agencies. Government core business is predominantly about delivering portfolio-specific services, such as roads, schools and health services. Government funding is structured to focus on the delivery of these services and so addressing equity comes to be seen as in addition to core business, rather than underpinning government business. Respondents explained the view that equity is optional and “a nice thing to do” rather than central to their core business, for example:

…equity is often seen as—I think from a moral perspective it’s valued but from a funding perspective it’s often seen as something that you do when you have additional funding to support a focus on equity. If your core business is buses and roads and trains and you’ve only got enough funding to deliver what you need to on buses and roads and trains, then really consideration of how effectively those bus routes meet the needs of low income earners in Salisbury is less important than actually making sure that the system’s running, so it starts to be one of those things that gets cut out.

(Interview 75, health sector, 2014)

I think the work in equity was always seen as another—“a nice thing to do” (…) but not a core thing, nor was there an outcome and so it was never actually—it was always to improve health and wellbeing etc. Equity was very rarely an anticipated goal or outcome and if it was it was never in the political environment anyway.

(Interview 74, health sector, 2014)

The small policy space given to equity meant that “inequity almost becomes invisible because you’ve got those big other political imperatives” (Interview 13, health sector, 2013). Thus the focus on equity by government is undermined by competing political agendas and imperatives. As a result equity was not a key driver of HiAP’s partners or of the government’s policy agenda more broadly.

There was an institutional shutting down of the already small policy space for health equity with the worsening state economic context. Actors appeared to have no ownership of equity. Equity was seen as ‘other’, not essential, and not their responsibility. While there were ‘mixed messages’ from government, the government mandate became increasingly focused on addressing economic priorities and job creation over the social policy priorities that came from the period pre-dating the State’s economic difficulties. The achievement of equity was seen by some respondents to be possible through an indirect trickle down approach:

…the equity objectives unfortunately tend to be the ones that are thought about a bit far down the track and are usually sort of like encapsulated “well, if we get economic growth, then everyone benefits and there you are, you’ve got your social objectives sort of like ticked off”.

(Interview 21, transport sector, 2013)

This understanding that equity can be achieved through an indirect trickle down of benefit as a result of addressing economic priorities reflects the dominant neo-liberal market ideology of many affluent countries, including Australia [54]. The understanding that economic priorities take precedence over equity was evident in comments from many respondents, for example respondents explained:

People talk about (equity) but in my experience if you come forward with a program whose sole purpose is to create equity then it isn’t going to resonate with the politicians. Even the politicians of the left are much more economically rational and focused on—you know—it’s the economy stupid, that sort of thing, so it does make equity problematic.

(Interview 79, health sector, 2014)

I thought the business of government is equity and any political manifesto has equity central to it, but there’s just a difference between the extent to which a political regime is going to be dependent upon the market to deliver that, to what extent that the re-distributional activities of government is going to deliver equity (…) Clearly as the budget cuts come in there’s a greater reliance on the market to deliver.

(Non-health sector Program Logic Model Workshop, 2013)

Similarly, the Health Minister at the time HiAP was implemented indicated that while equity was an important part of his policy platform, it was inevitably dominated by the “real political pressure” of budget cuts and overspends, waiting lists and increasing hospital demand [55]. 

#### 3.3.4. Implications of the Co-Benefits Approach

HiAP drew strongly on the assumption that intersectoral collaboration is necessary to bring about policy action on the social determinants of health. Its focus on process was intended to produce conditions that would in turn produce healthy public policy in keeping with the government’s priorities. Examples of collaborative partnerships established and developed by HiAP are included in Table 4, in particular in relation to the Health Lens Analysis projects. Specific concrete examples of these partnerships include: with the Education Department on engaging parents in their children’s literacy; with the Transport Department to improve Aboriginal road safety; and with a number of partner agencies to support the Health Department’s healthy weight agenda, for example by increasing walking and cycling opportunities, and encouraging the community to visit national parks and be physically active. 

Adopting a co-benefits approach by which other sectors achieve their core agenda and health is also advanced has been central to the way that HiAP has functioned in South Australia. Our respondents understood that a co-benefits approach enabled the HiAP team to engage with other agencies on their core business because they sought to put the other agencies’ priorities and interests first, “to be seen to be useful”. A respondent explained:

…we had prided ourselves on starting on the core business of other agencies, being very focused on public policy issues, not health policy issues, and through that way being able to find co-benefits and co-alignment between outcomes.

(Interview 84, health sector, 2014)

In doing this, the HiAP team also sought to increase the understanding of actors in partner agencies of the health impacts of their policy actions and consequently to address health priorities [4,56].

We found that the focus on co-benefits brought significant benefits to participants from other sectors who had been involved in HiAP activities, including by broadening their perspectives in relation to the health impacts of their agencies’ business. 

Consistent with this co-benefits approach, HiAP placed great importance on relationship maintenance with partner agencies as this was seen as crucial to engaging with them on HiAP initiatives. As a result a significant amount of time and energy was put into relationship establishment and maintenance in order for HiAP to undertake its cross-sector collaborative work, to the point that some respondents felt that the process took too long. As a consequence of the emphasis placed by the HiAP team on relationship maintenance and on working on partner agencies’ agendas (the co-benefits approach), and because the equity agenda did not “resonate” with partner agencies, the process goal of establishing and maintaining relationships was privileged over the equity outcomes and consequently equity became practically invisible in HiAP activity.

## 4. Discussion

While SA HiAP started with equity as part of its core principles, in practice this commitment has rarely moved beyond this initial rhetoric to practical application. HiAP’s goal of intersectoral action to address complex policy issues became the primary agenda for HiAP over its goal of improving equity. We have used the lens of institutional theory to examine these central findings. This enables us to examine why equity was not privileged as a policy idea and how actors, institutions and prior history have played a role in limiting HiAP’s focus on equity. 

The social determinants of health and health equity are policy ideas that underpinned the establishment of HiAP internationally, including in South Australia. In an international scoping review of HiAP in 2011, Shankardass, Solar et al. found that while all of their 16 reviewed examples of HiAP emphasised downstream health care interventions, less than one third of these emphasised upstream interventions such as income or power redistribution [57]. Similarly, despite having improving equity as one of its goals when it was established, in South Australia the focus of HiAP has shifted to be more on addressing the social determinants of health through intersectoral policy without an explicit focus on health inequities. 

Table 5 summarises our main research findings on the enablers and barriers to equity being progressed by HiAP in South Australia.

### 4.1. Equity Not Privileged as an Idea

Equity is a policy idea in the sense that it is a principled belief grounded in human rights and social justice [1,58]. As such it has the potential to serve as a road map and to guide policy action for significant social change, particularly if it becomes established as an institutional cultural norm. However, in the implementation of HiAP in South Australia, health equity has only occasionally been explicitly raised during the definition of policy problems. Many of the outputs of the HiAP Health Lens Analysis projects have successfully demonstrated links between the interests of the Health Department and those of partner agencies, and through HiAP processes, have raised the awareness of partner agencies about their role in promoting health through addressing the social determinants of health. Conversely, there has been a relative silence on the underpinning social determinants of health equity and on the existence of inequities. 

Our study shows how, despite equity (as it was variously understood), being seen by individual policy staff as an assumed and normal aim of government social policy, and despite it being evident in policy statements, it was not overtly part of the implementation of HiAP’s activities in South Australia. We have found a shift away from equity in HiAP in response to shifting government policy priorities. 

Despite respondents recognising equity as a principle of the government’s social policy agenda, and therefore comprising a central policy idea, the reality of other competing government priorities has meant that equity is seen as optional, rather than mainstream. This highlights the dilemma for public servants and politicians who may personally have a value base supportive of equity but are not able to act on this because of institutional constraints. These constraints limit even senior individual actors’ influence and actions [59]. As a result they report feelings of discomfort and dissonance in relation to their policy ideals and value base if these are supportive of equity and cannot be realised in the face of the neo-liberal institutional basis of the government system. 

Howlett, Ramesh and Perl explain that governments’ neo-liberal economic policy orientations constitute a meta-institution [45]. In South Australia this orientation appears to have overshadowed the government’s social policy agenda and to have limited and constrained policy actors’ ability to consider or prioritise policy ideas related to equity over or to the same extent as the economic policy agenda. Consistent with a neo-liberal economic perspective, we found evidence of an expectation that if economic concerns are effectively addressed the resulting “trickle down” of benefits would mean that no further action to address inequity would be required [60]. 

Howlett, Ramesh and Perl state that institutions can be creative, but are inherently stable and resistant to change [45]. In the case of the SA HiAP approach, the dominant neo-liberal policy agenda, which also determined how the government responded to the state economic crisis, shut down the “small policy space” that was available for equity, and underpinned and constrained government thinking and priority setting [18]. The government exhibited a sense of panic about the perceived economic crisis and reacted in a way that appeared to be primarily concerned about maximizing the likelihood of re-election by prioritizing economic development opportunities and short term job creation strategies over longer term social policy considerations including equity [4]. This phenomenon, described by Aidt and Dutta as policy myopia, explains “short termism” in policy, a focus on short term political wins over a progressive focus on the future [61]. 

Policy actors generally saw the concept of equity positively, and it was evident in SA Government policies, for example as a part of the definition of wellbeing used in the *South Australia: State of Wellbeing* document (see Table 4). However we found very few examples of strategies to promote equity being operationalised during the implementation of HiAP in South Australia. Hunter, Popay, Tannahill and Whitehead [19], and Rigby and Hatch [18] identify key impediments to addressing equity which include lack of political will and a siloed framing of agency core business. Hunter, Popay, Tannahill et al explain that while policies often commence with broad statements recognising the social determinants of health and health equity, implementation shows a drift towards more concrete individual/behaviour-based lifestyle responses and a focus on disadvantage. They argue that more concrete responses have a greater likelihood of being adopted by governments because they are easier to implement than controversial and politically challenging responses, such as those required to address inequities [62]. Dahlgren and Whitehead explain that political will to address equity requires a political commitment and desire for redistribution of resources to address inequity, and a commitment to working across organisational silos and single portfolio funding to achieve this [12]. Consistent with this preference for less complex and more concrete policy responses, the core business of government is defined by its agencies according to narrow siloed portfolio-based foci with resources and responsibilities allocated within these silos, creating challenges for progressing an equity agenda. 

Exworthy [63] discusses the challenges for policy makers in implementing policy responses to the social determinants of health and health equity. These challenges arise from a number of factors including the lack of identified “simple problem solutions”, the dominance of other competing priorities, and the gaps in knowledge and understanding of the causal pathways which result in a focus on process measures over outcomes [63]. Our data show that policy actors found equity difficult to conceptualise and actors from different sectors understood and interpreted equity differently. As a result, health policy actors made conscious decisions not to raise equity with partner agencies, and their decisions were consistent with the government’s priorities which shifted to overwhelmingly focus on their economic agenda at a time of perceived crisis. 

While the importance of equity as a policy idea was recognised by many of our respondents, equity did not become an idea embedded within the political and bureaucratic institutions that drive policy. The Health Lens Analysis approach meant that HiAP operated at a project level and worked within departments on their priority issues rather than it being systematised (which now may be occurring through the implementation of the institutional mechanism of public health legislation, although this is still to be confirmed, see Table 4). 

Because equity was not institutionally embedded, and ideas about its importance did not pervade discourses beyond HiAP projects, and only to a very limited extent within these projects, it could be dismissed as “outside” rather than as a central part of core business. Equity remained an individually held value/ideal of some and not a collectively held institutional value that drove government business. 

### 4.2. The Role of Actors

HiAP has two central aims—to address the social determinants of health and health equity through intersectoral policy development; and to promote and build capacity for intersectoral collaboration for shared policy goals [31]. The focus of HiAP activity in South Australia was determined by its authorising environment, driven initially by the SASP priorities which included equity targets as well as a focus on working across government to achieve these targets [4]. 

In South Australia, an emphasis on relationships was critical to the HiAP team gaining the trust and collaboration of other agencies. Our data show that a co-benefits approach was effective in creating opportunities for HiAP engagement and was valued by partner agencies. This is a positive process outcome from this study which has been discussed in detail in another paper [64]. However, this approach also meant that HiAP activity started with the priorities of the partner agency. Therefore unless the partner agency’s priorities coincided with the goals of the Health Department, health goals were likely to receive less attention than the goals of the other sector. Ollila has identified four strategies for policy-making through HiAP including the ‘co-operation strategy’ (or co-benefits approach) [33]. We have found that while this strategy has significant benefits for engaging with partners and gaining their commitment to participating in HiAP activity, the implication of privileging relationships was that HiAP accepted and worked with the other agency’s priorities. In doing this, explicit strategies to address equity were lost.

In our analysis of SA HiAP, as part of developing a program logic model for this research, we positioned intersectoral collaboration as a process goal that could contribute to the achievement of equity, and we recognised the importance of intersectoral collaboration in HiAP’s work to engage partner agencies to bring about changes in equity [42,65]. However, given the lack of attention to equity as a focus in these intersectoral collaborative processes, HiAP as it is currently implemented in SA is unlikely to bring about significant change in health equity.

Graham highlights the distinction between the social determinants of health and the social determinants of health equity and argues that focusing on social determinants of health without considering the social processes that determine them can result in persistent inequities despite positive trends in health and health determinants [13]. Following involvement with HiAP, partner agencies reported a greater understanding of the social determinants of health, and of how their agencies contributed to these. HiAP activity explicitly articulated the links between health interests and those of their partner agencies, and explicitly identified the social determinants of relevance to their partners. There is a clear difference in the approach HiAP adopted in raising and identifying the social determinants of health in general as they related to partner agencies’ business, and the social determinants of health equity specifically, which were not made visible to HiAP’s partners. While HiAP explicitly argued the link to the social determinants of health, it was complicated to take this argument further to the social determinants of health equity. Exworthy and Hunter support this finding when they explain that health inequities are complex, multifaceted wicked problems with no apparent simple solution and so getting health inequity on to the government policy agenda is difficult as a result of competing priorities and a preference for concrete and less controversial solutions [32]. 

The constraints on progressing an equity agenda can be seen as being constructed through the limitations created by the institutions of the government’s key strategic documents and the neo-liberal values of privileging the economic agenda over the social policy agenda [32]. While the government focus on equity, evident in the SASP targets (see Table 3), slipped with the worsening economic climate, its intersectoral joined-up policy agenda was sustained under the institutional values of public sector reform and innovation which continued to be promoted actively by the government. As a result of its focus on intersectoral policy development through a co-benefits approach, HiAP was primarily viewed by policy actors as an innovative government initiative for doing joined-up policy (rather than for addressing equity), which as a policy idea had increasingly become an additional institutional focus in South Australia in relation to public sector values and behaviour. This array of reasons—the perception among policy actors that equity is complex and difficult to address; the focus of HiAP on co-benefits, which resulted in a silence on equity; and the strong government interest in joined-up policy solutions, can be understood as multilayered reasons for a shift from the goal of equity as an outcome to a focus on process. These multilayered reasons are underpinned by the meta-institution of neo-liberalism, whereby the primary role of the state is seen to be the efficient functioning of the market and so economic priorities are dominant [66].

### 4.3. The Role of Institutions and History

Cairney explains that institutions include established practices, standard operating procedures and relationships that produce regular patterns of policy-making behaviour [44]. As discussed previously, Hunter, Popay, Tannahill et al. describe the slide from broader consideration of social determinants of health and health equity in policy rhetoric towards individual/behavioural solutions in policy implementation that target the most disadvantaged [62]. It was evident in our research that the new policy idea of the social gradient was unfamiliar and harder for policy actors to understand and act on. Our analysis of the way that interview respondents problematised the concept of equity highlights that the interpretation of equity in the implementation of HiAP was framed in familiar terms—in particular as a focus on vulnerable groups. This is a well-traversed policy terrain for the public sector which has a long history of providing portfolio-specific services in response to policy issues focused on vulnerable groups. The problematisation of equity as being about vulnerable groups therefore provides a tangible, familiar and concrete way for agencies to respond. 

Neo-liberalism, like other policy ideas, becomes institutionalised when it is assumed and not questioned or challenged [67,68]. The policy position on promoting equity by working with vulnerable groups appears to be entrenched, limiting the potential for policy action to take a different approach such as levelling the gradient through universal strategies [32]. This phenomenon has been described as “path dependency” [69]. Considine provides a useful analogy here when he describes the influence of the way policy issues have been thought about and acted upon in the past as being like “ruts in the road” that are well worn and very hard to leave, and “provide both a short cut and a constraint for those who follow” [60] (p. 2). Policy is built on what went before, embedded in routine practices and following proven habits and customs, with little being entirely new. Policy action is therefore institutionally framed [32,60]. 

Equity is a distal and contested concept that requires a systemic consideration of issues of power and control, and a deep analysis of the underlying causes [59]. In contrast, intersectoral collaboration is a familiar process with a long history of public sector attention. Our data show that for many respondents, HiAP has come to be seen as a means to achieve intersectoral collaboration through following a clearly defined series of steps using the Health Lens Analysis approach. This is congruent with Carey’s idea that HiAP is an instrumental process-based intervention, the implementation of which may be instrumental in creating policy that improves health, but is not, as an intervention, inherently able to improve health [70,71]. The Health Lens Analysis process sought to build causal pathways to demonstrate links between other sectors’ work and health, but explicit causal pathways were not explored between other sectors’ work and health equity, and without this the potential to develop equitable policy was not being built.

As well as being constrained by neo-liberalism and a preference for relationship maintenance, HiAP was unlikely to make a significant impact on improving equity in South Australia because it operates within a very small budget. With more resources, HiAP may have been able to work with local government to consider their role in equity, or to engage outside the public sector with groups such as Aboriginal communities and others living in vulnerable situations about what equity means. There is an opportunity for local government to progress an equity agenda in their regional public health planning exercises. Similarly, while it is still early days for the Public Health Partner Authorities, there is potential for these partnerships to advocate for and progress an equity agenda; and if it adopts a genuine equity approach supported by a strengthened social policy agenda, the government statement—*South Australia: State of Wellbeing* has the potential to put equity explicitly on the public policy agenda, increasing the chance of equity gaining traction as a government social policy priority. Further opportunities to address equity and promote it as a key policy idea were evident in our data and could have been pursued by HiAP if more resources were available for them to do so.

There is evidence of shifts internationally which may signal change. For example, the recent adoption by G20 governments of the United Nations led *Transforming our World: the 2030 Agenda for Sustainable Development* may create a new international opportunity to frame discussions around equity differently through the lens of sustainability, the core idea of the Sustainable Development Goals [72]. Although they have been criticised for being aspirational [73] and not dealing with some of the underpinning structural inequities or questioning the neo-liberal growth paradigm [74], the Sustainable Development Goals are underpinned by the principles of equity and justice, and promote a Health in All Policies approach through advocating greater policy coherence across sectors [75]. Similarly, the International Monetary Fund has recently released a report examining the impacts of tax cuts which found that lowering tax rates for the rich stimulates the economy, but also increases inequality within society [76]. Such international developments may create new opportunities for the equity agenda to gain traction as a key policy idea.

## 5. Conclusions

Tackling inequities poses dilemmas for government and policy makers as it requires challenging existing powerful political and economic interests as well as dominant ideologies. Framing equity as being about vulnerable groups and their access to services gave some tangible and familiar parameters to equity as a distal and controversial concept for HiAP in South Australia, and helped partner agencies understand how they and the Health Department could contribute to particular issues that were of mutual interest and benefit. However, framing equity in this way removed the focus on the fundamental ways that power and social structure create inequities. 

Our research found that beyond broad policy statements, the focus of HiAP in South Australia was never really on equity. This paper describes a number of deep institutional barriers to addressing equity, in particular the dominance of neo-liberalism.

Equity is a distal goal and so the work of SA HiAP could only show evidence of an equity impact over the coming decades, as predicted in the Program Logic Model developed for this research [65]. Realistically, HiAP was unlikely to improve equity in South Australia given the context of the adverse economic environment in which it was operating and the institutional constraints on its capacity to work on equity explicitly. Because it was not an explicit focus of the HiAP team in their work with other agencies, equity is unlikely to translate to a priority for these agencies. The strategies that SA HiAP has pursued to date have not been designed primarily with the intention to address inequities explicitly, and so it is unlikely that they will improve equity.

The priority for HiAP became increasingly about joined-up policy and collaboration in response to the government’s public sector reform agenda, the government’s narrowing policy priorities, and in order to establish and build relationships and identify co-benefits to engage other agencies. While improving health, wellbeing and equity, and intersectoral collaboration were the dual goals of HiAP, facilitating collaboration to address complex intersectoral policy issues became its dominant goal, with equity gaining little traction. 

## Figures and Tables

**Table 1:** Inequality rate ratio for Australia and South Australia ^1^.

Area	1987–1991	2010–2014
Australia	1.55	1.90
South Australia	1.57	2.05

^1^ Source: Public Health Information Development Unit, Torrens University, South Australia [26].

**Table 2:** Equity focus of key strategic priority and direction setting documents.

Document	Focus on Intersectoral Collaboration	Focus on Vulnerable Groups	Focus on Access to Services	Focus on Closing the Gap/Equity
Health in All Policies—The 10 Principles (2007)				
South Australia’s Strategic Plan (2007 & 2011 versions)				
7 Strategic Priorities (2011)				To a limited extent (in relation to early years priority only)
10 Economic Priorities (2014)				

**Table 3:** Examples of equity-focused targets in the 2007 and 2011 versions of *South Australia’s Strategic Plan*.

SASP—2007 Version	SASP—2011 Version
**T6.5 Economic disadvantage:** Reduce the percentage of South Australians receiving government benefits (excluding age pensions) as their major income source to below the Australian average by 2014.	**16. Economic disadvantage:** By 2020, increase by 2 percentage points the share of total household income earned by low income South Australians.
**T2.4 Healthy South Australians:** Increase the healthy life expectancy of South Australians by 5% for males and 3% for females by 2014.	**78. Healthy South Australians:** Increase the healthy life expectancy of South Australians to 73.4 years (6%) for males and 77.9 years (5%) for females by 2020.
**T2.5 Aboriginal healthy life expectancy:** Lower the morbidity and mortality rates of Aboriginal South Australians.	**79. Aboriginal healthy life expectancy:** Increase the average healthy life expectancy of Aboriginal males to 67.5 years (22%) and Aboriginal females to 72.3 years (19%) by 2020.

**Table 4:** Health Lens Analysis (HLA) research case studies and subsequent HiAP-based work

Case Study	Focus	Evidence of Health Equity in Proposal	Evidence of Health Equity in Project Outcomes
**Health Lens Analysis projects**			
Raising parental engagement with literacy to improve literacy outcomes for children in the early years of schooling HLA	To investigate how to better engage families from disadvantaged backgrounds in creating a literacy rich environment for children at home and school	√ Although focus of HLA project was on 4 schools in lower socio-economic status communities, the proposal included evidence of a broader health equity aim	√ Development of resources for all Education Department schools to support HLA aims, although the subsequent department restructure may have changed this focus
International students’ health and wellbeing HLA	To address the gap in information provision and support provided to international students, focused on international students in the Vocational Education and Training sector who do not have access to the same support as university students	X Focus on improving the health and wellbeing of international post-secondary school Vocational Education and Training students studying in SA, and their knowledge of and access to health services	X Final output of the project was a publication focused on how international students could look after their individual health needs and providing details of available services
Healthy weight HLA	To increase commitment across government to actively support the healthy weight agenda by identifying policy opportunities for a range of government departments to support the achievement of the SASP Healthy Weight target	X Focus on identifying other agencies that could act on the healthy weight agenda and on meeting their agendas/core business	√ Evidence of health equity in Eat Well Be Active Strategy (2011–2016)
Healthy sustainable regional development HLA	To identify mechanisms and strategies to improve the health, sustainability and economic positioning of communities in the Upper Spencer Gulf so that they can capitalise on opportunities presented by the proposed expansion of the resources sector in the region	√ Strong focus on “triple bottom line” including social goals and specific reference to equity	√ Developed a Regional Atlas of Community Wellbeing with focus on inequities and social determinants of health for regional planning purposes
Aboriginal road safety HLA	To collaboratively identify ways to increase Aboriginal healthy life expectancy by improving road safety through increasing safe mobility options, focusing on drivers’ licensing and diversionary programs that support Aboriginal people to obtain and retain their drivers’ licences in remote South Australia	√ Explicit reference to health equity in proposal and project aim	X Experienced difficulty developing recommendations all agencies would sign up to. No mention of equity in final recommendations
**Public health regulation and planning**			
*South Australian Public Health Act 2011*	Legislation to promote and protect the health of South Australians through a public health approach	√ Equity reflected as a principle in the Bill introduced into Parliament	√ Equity is a principle in the South Australian Public Health Act 2011.
Regional public health planning by local government	To achieve the objects of the Act consistent with the State Public Health Plan	? Public information unavailable	The State Public Health Plan includes a strong focus on equity.Regional public health planning by local government currently includes a focus on social determinants of health, although this may change over time.
Establishment of Public Health Partner Authorities	Developing agreements with government and non-government agencies committed to working on the achievement of State Public Health Plan priorities at a state or regional level	? Public information unavailable	X The work to establish Public Health Partner Authorities currently focuses on collaboration between agencies to improve population health and wellbeing although this may change over time.
**90-Day Projects led by HiAP**			
Working together for joined-up policy delivery	To develop tools and guidance for working intersectorally across government to ensure that collaboration becomes a greater focus of government policy and practice	? Public information unavailable	X Focus on intersectoral collaborative policy development
SA: State of Wellbeing (developed a whole of State Government statement on wellbeing)	To contribute to the development of an agreed description and position on wellbeing in the South Australian context through development of a whole of State Government statement on wellbeing	? Public information unavailable	The definition of wellbeing in *South Australia: State of Wellbeing Statement* includes reference to equity as a factor, although this is not further incorporated into the Statement

**Table 5:** Summary of findings on enablers and barriers to progression of equity by SA HiAP.

Key Elements of Institutional Theory	Enablers	Barriers
Ideas	- Equity accepted by policy actors as legitimate public policy concern Statement on the importance of equity evident in policy documents	- Dominance of economic priorities over social policy ideas ‘Trickle down’ economic benefits expected to address equity without further action Equity not seen as core business Equity evident in some policy statements but shift to concrete focus on lifestyle/individual behaviour in strategies Lack of shared understanding of equity
Actors	- Policy actors have value base supportive of equity Policy actors feel able to act on policy statements concerning equity	- Lack of political will to deal with fundamental social structural issues Policy actors operate within constraints of government institutional priorities Focus on social determinants of health in intersectoral policy, with equity remaining implicit and invisible Co-benefits prioritise partner agencies’ agendas over health agenda
Institutions	- SASP provided initial mandate and focus for SA HiAP and included some equity focus Government intersectoral policy agenda driving public sector focus on complex problems (potential for this to include equity)	- Neo-liberalism an overarching meta-institution Shift in government priority setting documents away from equity Siloed framing of agency core business Process focus on intersectoral collaboration preferenced over outcome focus on equity

## Abbreviations

DPC	Department of the Premier and Cabinet
HiAP	Health in All Policies
HLA	Health Lens Analysis
SA	South Australia
SASP	South Australia’s Strategic Plan
